# A novel X-linked mutation in *IL2RG* associated with early-onset inflammatory bowel disease: a case report of twin brothers

**DOI:** 10.1186/s13256-023-04049-y

**Published:** 2023-07-18

**Authors:** Elham Rayzan, Mona Sadeghalvad, Sepideh Shahkarami, Samaneh Zoghi, Zahra Aryan, Seyed Alireza Mahdaviani, Kaan Boztug, Nima Rezaei

**Affiliations:** 1 International Hematology/Oncology of Pediatrics’ Experts (IHOPE), Universal Scientific Education and Research Network (USERN), Boston, MA USA; 2 Research Center for Immunodeficiencies, Pediatrics Center of Excellence, , Children’s Medical Center Hospital, Tehran University of Medical Science, Dr Qarib St, Keshavarz Blvd, Tehran, 14194 Iran; 3 Department of Immunology, School of Medicine, Tehran University of Medical Sciences, Tehran, Iran; 4 Dr. Von Hauner Children’s Hospital, Ludwig-Maximilians-University Munich, Munich, Germany; 5 Medical Genetics Network (MeGeNe), Universal Scientific Education and Research Network (USERN), Munich, Germany; 6 Ludwig Boltzmann Institute for Rare and Undiagnosed Diseases, Vienna, Austria; 7 Department of Emergency Medicine, Massachusetts General Hospital, Harvard Medical School, Boston, USA; 8 Pediatric Respiratory Disease Research Center, National Research Institute of Tuberculosis and Lung Diseases (NRITLD), Shahid Beheshti University of Medical Sciences, Tehran, Iran; 9 Department of Pediatric Hematology and Oncology, St. Anna Children’s Hospital, Department of Pediatrics, Medical University of Vienna, Vienna, Austria

**Keywords:** SCID, Immunodeficiency, Case report, IL2RG, Inflammatory bowel disease

## Abstract

**Background:**

X-linked severe combined immunodeficiency is caused by *IL2RG* gene mutation. Several variations have been identified in the *IL2RG* gene, which potentially can prevent the production of nonfunctional proteins. Herein, a novel X-linked variant in the *IL2RG* gene is reported in twin brothers, associated with inflammatory bowel symptoms.

**Case presentation:**

The patients were 26-month-old monozygotic twin middle-eastern males with failure to thrive and several inpatient admissions due to severe chronic nonbloody diarrhea that started at the age of 12 months. Pancolitis was revealed after performing upper and lower gastrointestinal endoscopies on the twin with more severe gastrointestinal symptoms. Flow cytometric evaluation of the peripheral blood cells showed low levels of CD4+ cells in both patients. Next generation sequencing-based gene panel test results of the two patients proved a novel heterozygous missense X-linked *IL2RG* mutation (70330011 A > G, p.Trp197Arg) in one of the patients, which was predicted to be deleterious (CADD score of 28), which soon after was confirmed by Sanger segregation in his twin brother. Both parents were wild types and had never experienced similar symptoms. The patients received an human leukocyte antigen (HLA)-matched cord blood transplant. The twin with more severe gastrointestinal symptoms died 1 month after transplantation. In his brother, watery diarrhea eventually subsided after transplantation.

**Conclusion:**

Intestinal involvement in X-linked severe combined immunodeficiency is a rare presentation that might be neglected. The increasing availability of genetic screening tests worldwide could be helpful for early detection of such lethal primary immunodeficiency diseases and in implementing effective interventions to handle the severe outcomes.

## Background

X-linked severe combined immunodeficiency (X-SCID) is a life-threatening disease involving both the cellular and humoral immune systems. This immune disorder is considered the most common form of SCID and is caused by mutations in the interleukin-2 gamma chain or *IL2RG* gene (γc chain) resulting in the absence of T and natural killer (NK) lymphocytes as well as nonfunctional B lymphocytes [1]. The γc chain gene encodes an important component of the receptors for cytokines including interleukin-2 (IL-2), IL-7, IL-9, IL-4, IL-21, and IL-15 [2]. Therefore, mutations in γc lead to disruption of the signal transmission associated with the cytokine receptor as well as lymphocyte development.

X-SCID is almost frequently lethal in the first 2 years of life until the immune system is reconstituted by bone marrow transplantation or gene therapy [1]. X-SCID usually manifests in early childhood between the ages of 3 and 6 months with recurrent infections, failure to thrive (FTT), chronic diarrhea, absent tonsils, and lymph nodes. Furthermore, children with SCID are more likely to become infected with community-acquired infections, so early diagnosis is essential [3].

Because of the gene location on the X chromosome, all cases with X-SCID are males and mothers could be carriers of the pathologic variant on the X chromosome. There are two important variants of the IL2R including null and hypomorphic variants resulting in typical and atypical X-linked SCID, respectively [4]. Mutations in the *IL2RG* gene causing X-linked SCID were identified for the first time in 1993, and since then more than 200 mutations in the *IL2RG* gene have been recognized with the highest frequency of frameshift mutations [5, 6]. Absent γc signaling in typical X-SCID leads to absent peripheral T and NK cells and dysfunctional B cells, while partial impairment of γc signaling in hypomorphic variants leads to a milder form with variably reduced numbers and/or function of T, B, and NK cells [7]. In contrast to the typical X-SCID which is fatal, patients with hypomorphic variants can live untreated and might develop autoimmune disorders along with recurrent infections [3].

The clinical phenotypes of X-SCID are poorly described, and this could be the reason why these patients are usually diagnosed late in childhood or even in adulthood as well as delay in their treatment. As a result, timely genetic evaluation and implementation of effective therapies can improve symptoms and reduce morbidity in these patients. Herein we describe a novel mutation in the *IL2RG* gene in twin brothers associated with early-onset inflammatory bowel disease. Timely genetic screening of individuals with the same clinical symptoms could help in early diagnosis and the implementation of effective treatment to handle severe outcomes.

## Case presentation

**Fig. 1:**
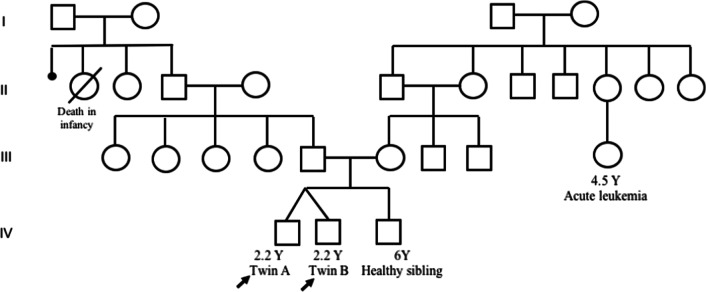
Pedigree of the patients. The patients were heterozygous for *IL2RG* mutation (70330011 A > G, p.Trp197Arg), shown by a solid black symbol

Twenty-month-old monochorionic diamniotic twin middle-eastern brothers born to non-consanguineous parents (Fig. 1) were referred to Children’s Medical Center following chronic nonbloody diarrhea that started at the age of 12 months. Family history was positive for acute leukemia in one of the relatives, and for the death of their paternal grandparents’ daughters due to unknown reasons during infancy. Their older 7-year-old brother and both parents were healthy.

**Fig. 2:**
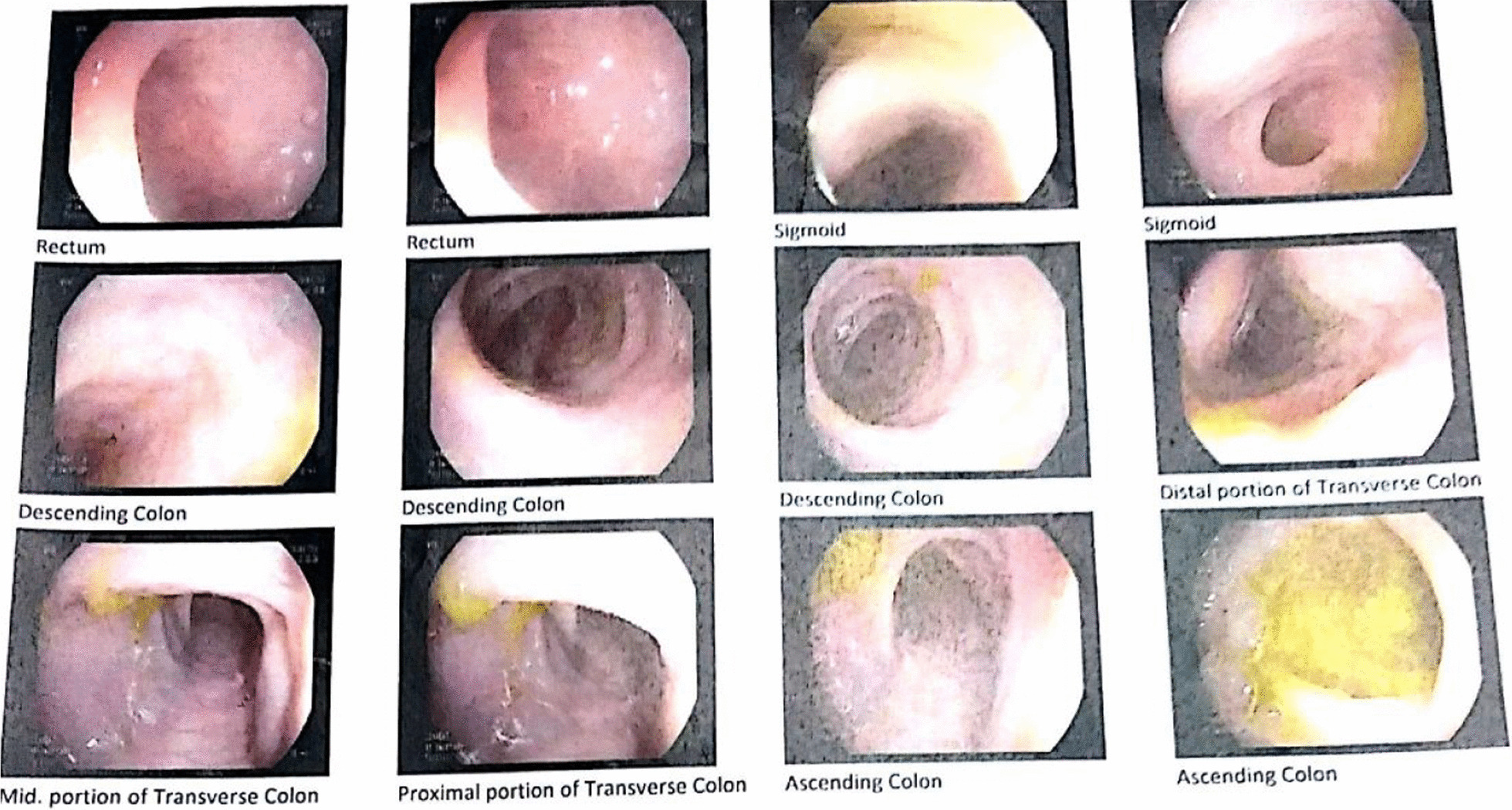
Lower gastrointestinal endoscopy (twin A) revealed diffusely abnormal vascular and mucosal pattern in rectum, sigmoid, descending, transverse, ascending colon, and cecum

General examinations showed normal chest, head, ears, eyes, nose, and throat (HEENT) evaluation, abdominal, and extremity examination. The twins’ initial hospitalization occurred when they were 2 months old owing to concurrent pneumonia, which affected both infants within a brief timeframe. They were brought to the emergency room exhibiting symptoms of high fever (38.8 °C) and poor feeding. The decision to admit them to the hospital was based on the severity of their symptoms and elevated levels of inflammatory markers. At the time of admission, review of the systems and physical examinations were normal except for respiratory symptoms including subcostal retraction, tachypnea, and diffused crackles in the lung fields. It is important to mention that, other than high fever and mild tachycardia, vital signs were within normal ranges. Intravenous administration of cefotaxime was initiated, and after undergoing 5 days of treatment and close observation, the twins were discharged from the hospital. When they were around 12 months of age, they experienced nonbloody nonfatty diarrhea that became chronic. Gastrointestinal symptoms were more severe in one of the brothers (twin A), so upper and lower endoscopy was performed. The results of the endoscopy showed normal esophagus and stomach but diffuse nodularity in the bulb and the second part of the duodenum. The result of the colonoscopy (Fig. 2) showed multiple aphthous lesions and ulcers in the rectum, sigmoid, descending transverse, ascending colon, and cecum. The vascular and mucosal patterns were abnormal, and he was diagnosed with pancolitis. In addition, his radiological findings revealed duodenal and jejunal mural thickening. He was evaluated for cytomegalovirus (CMV) and Epstein–Barr virus (EBV), and also for *Mycobacterium*. The polymerase chain reaction (PCR) on the colon tissue sample confirmed CMV colitis, and the patient received intravenous ganciclovir, metronidazole, soluvit, cefotaxime, and pantoprazole. The tissue sample was negative for *Mycobacterium* evaluated by PCR. Further investigations confirmed positive Epstein–Barr virus serology. However, the other brother’s tests were negative for the mentioned viral infections. In addition, herpes simplex virus (HSV) PCR of cerebrospinal fluid was negative in both patients. Their upper intestinal pathology results showed variable villous atrophy and increased lamina propria chronic inflammation along with some neutrophils and eosinophils in the duodenal mucosal biopsy. Crypts showed mild hyperplasia and increased mitosis. The endoscopy and pathology findings were interpreted by an expert gastroenterologist to finalize the diagnosis of IBD and rule out other causes.

Both of the brothers had experienced recurrent respiratory symptoms and two episodes of otitis media that were treated with oral antibiotics without notable complications. They had failure to thrive and three or four hospital admissions due to severe watery diarrhea. Despite being in the lower-than-normal percentile for weight, the twins’ physical examinations including head circumference and childhood milestones were preserved normal for their age. Moreover, complementary investigations demonstrated G6PD deficiency in the twins, which was in accordance with episodes of jaundice and hemolytic anemia. The twins’ vaccination status was up to date till the age of 1 year old. It is worth mentioning that, even though they received Bacille Calmette-Guérin (BCG) immunization during their neonatal period, they did not present any signs of BCG lymphadenitis or dissemination later in the disease course.

Laboratory investigations including complete blood count (CBC), immunoglobulin levels, flow cytometry for immunophenotyping, complement system evaluation, and CSF analysis including PCR HSV were performed. Besides, next-generation sequencing (NGS)-based primary immunodeficiency disease (PID)-gene panel screen, which targets > 500 genes in parallel, and Sanger sequencing were requested to investigate gene mutations.

The laboratory results are summarized in Tables 1 and 2. CBC results showed high WBC and platelet counts in both brothers. Immunophenotyping using flow cytometry showed decreased levels of CD4 and CD4/CD8 ratio, while CD19 and CD20 were normal in the two brothers. CD16 and CD56 were in the normal range for two brothers, except for the decreased level of CD16 in one brother (twin A). The immunoglobulin levels including IgM, IgG, IgA, IgE, and complement system evaluation including the level of C3 and C4 were in the normal range for the two brothers. No CNS infection was documented, and PCR HSV was negative for both patients.

**Table 1:** CBC results of the patients

	Patient		
	Twin A	Twin B	Normal range
WBC (× 10^3^/µL)	16.4	14.9	4–11
Neut (%)	48	28	38–80
Lymph (%)	40	60	18–50
Monocyte (%)	5	8	2–10
RBC (× 10^12^/L)	4.4	4.11	3.12–7.3
Hb (g/dL)	9.8	11.4	14–17
HCT (%)	34.4	33.5	41.5–50.4
MCV (fL)	78.2	86.4	80–96
MCH (pg)	22.3	27.7	27.5–33.2
MCHC (g/dL)	28.5	32.1	33.4–35.5
Plat (× 10^9^/L)	912	852	150–450

*WBC* white blood cells, *Neu* neutrophils, *Lymph* lymphocytes, *RBC* red blood cells, *Hb* hemoglobin, *HCT* hematocrit, *MCV* mean cell volume, *MCH* mean corpuscular hemoglobin, *MCHC* mean corpuscular hemoglobin concentration, *Plat* platelets

**Table 2:** Summary of laboratory evaluation

	Twin A		Twin B	Normal range
CSF HSV (PCR)	Negative		Negative	–
CMV (IgM)	0.3		Negative	< 0.85
CMV (IgG)	208		Negative	< 6
EBV VCA IgM	1.89		Negative	< 0.5
EBV VCA IgG	3.65		Negative	< 0.75
HIV AB	Negative		Negative	–
C3	0.71		0.9	0.89–1.87
C4	0.27		0.35	0.165–0.38
Immunoglobulins				
IgG (g/L)	7.6		9.2	6.58–18.37
IgM (g/L)	0.49		0.9	0.4–2.5
IgA (g/L)	0.52		1.1	0.8–3.5
IgE (IU/mL)	33		29	< 144
Isohemagglutinin, Anti A, IgM	1/4		1/16	
Isohemagglutinin, Anti B, IgM	1/8		1/32	
Blood flow cytometry analysis: lymphocytes subsets (%)				
CD3	44	72		35–78
CD4	5	4		22–62
CD8	36	64		12–36
CD19	14	15		3–14
CD20	14	16		3–15
CD16	1.36	4		3–17
CD56	25.5	11.5		3–17
CD4/CD8	0.13	0.06		1–3

*HSV* herpes simplex virus, *CMV* cytomegalovirus, *EBV* Epstein–Barr virus, *Ig* immunoglobulin

**Fig. 3:**
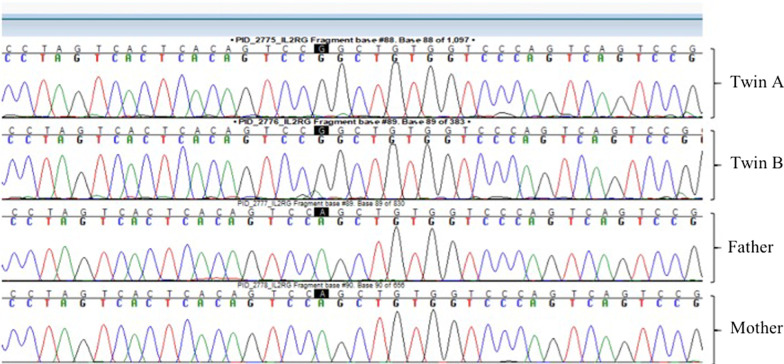
Sanger sequencing of the patients, and their parents. A heterozygous missense mutation in *IL2RG* (70330011 A > G, p.Trp197Arg) was shown in the patients. Both parents were wild type

Next-generation sequencing-based gene panel tests of the two patients proved a novel heterozygous missense X-linked *IL2RG* mutation (70330011 A > G, p.Trp197Arg) in one of the patients that was predicted to be deleterious (CADD score of 28), which soon after was confirmed by Sanger segregation in his twin brother (Fig. 3). Both parents were wild type and had never experienced similar symptoms. The patients received an HLA-matched cord blood transplant. Twin A, who was transplanted at the age of 3 years 6 months, died on day 25 post-transplant following a severe pulmonary infection. Watery diarrhea eventually subsided in twin B (the twin with less severe gastrointestinal symptoms) after transplantation at the age of 4 years 3 months. The post-transplant period was free of serious infections. On the basis of the most recent follow-up he is 7 years old with weight and height appropriate for his age. He has not experienced chronic graft versus host disease symptoms and is not taking immunosuppressants anymore.

## Discussion

We describe herein a novel missense mutation in the *IL2RG* gene in twin brothers. Our patients presented with FTT and inflammatory bowel disease since the age of 12 months. The patients developed chronic diarrhea in early infancy which could dictate early investigation regarding the possibility of primary immunodeficiency.

The *IL2RG* gene encodes the important component of the IL-2 receptor (IL-2R) known as γc. This protein forms the complete IL-2R along with IL-2Rα and IL-2Rβ. The signaling pathway originating from IL-2R plays a fundamental role in the development of T lymphocytes, especially regulatory T cells, and in preserving peripheral immune tolerance. Therefore, mutations in the *IL2RG* gene could disrupt lymphocyte development or cause functional impairment [1, 2].

Our patients had a c. 70330011 A > G substitution which leads to a p.Trp197Arg in the protein structure. Considering the monozygosity of the twins, our finding could suggest somatic germline mosaicism in the twin’s mother or an early post-conception mutation that happened before the embryonal separation. Immunophenotyping revealed a very low level of CD4 lymphocytes and a decreased CD4/CD8 ratio. The markers for B lymphocytes including CD19 and CD20 and the levels of immunoglobulins were in the normal range. On the other hand, the normal range of CD16 and CD56 indicated the presence of NK cells in both brothers. Altogether, this corresponded to a phenotype of T^−^ B^+^ NK^+^, which is uncharacteristic of an X-linked SCID phenotype. Typically, *IL2RG* mutations lead to a T^−^ NK^−^ phenotype. So, the normal frequencies of NK cells in these patients could be controversial. In IL2RG deficiency, NK cells of maternal origin have been identified. Estevez *et al.* in 2014 reported an 8-month-old boy who had recurrent diarrhea and dehydration. The genetic assessment showed an ACC insertion in exon 5 of IL2RG and phenotype of T^−^ B^+^ NK^+^, and the maternally derived NK cells were shown in this patient [8]. Furthermore, the normal numbers of NK cells are also reported in some variants of IL2RG. In this regard, the R222C mutation in *IL2RG* was shown to be related to normal levels of NK cells in SCID patients [9]. Additionally, a patient who had a mutation in exon 5, c.691G > A, R226H, had elevated levels of CD56+ NK cells [10]. Taken together, these data suggest that the presence of NK cells in IL2RG deficiency could be associated with the maternal origin of NK cells. Therefore, more studies are required to characterize the relation between IL2RG variants and NK cell frequency.

Our patients were admitted to the hospital for the first time at the age of 2 months because of pneumonia. Severe diarrhea developed at about 12 months of age. Infants with X-SCID become more susceptible to infection as the transplacental transmission of maternal serum antibody concentrations decreases. Medical care is sought for the majority of children between the ages of 3 and 6 months. However, presenting with a life-threatening illness before the age of 3 months is not rare. Other features, including FTT, recurrent infections, diarrhea, and pneumonia, may all result from a delayed diagnosis [3].

The twin brothers experienced IBD in early infancy. X-SCID affecting the gastrointestinal tract has been only reported in a 6-month-old boy who was identified with a point mutation in the *IL2RG* gene (c.536_552delTGAACCACTGTTTGGAG; p.Leu179Argfs*26), representing the T^–^B^+^NK^–^ phenotype of SCID [11]. The fundamental mechanism of intestinal involvement in X-SCID is little understood. CD4+ T cells coordinate effective immune responses, retain immune tolerance, and participate in memory cell differentiation [12, 13]. According to mouse model studies, the colonic microbiota is critical for recruiting an appropriate level of CD4 Foxp3-expressing regulatory T cells (Treg) to the colon to inhibit inflammation [14]. This implies that IBD could be caused by a lack of Treg-mediated immunity to commensals in the GI tract. Since the signaling of IL-2R plays a critical role in Treg development, the impairment of γc could result in reducing Treg development and infiltration [15]. Therefore, reduced infiltration of Tregs could be associated with a higher inflammatory response in the colon microenvironment.

Very early onset IBD (VEO-IBD) is diagnosed in patients with confirmed IBD findings who are younger than 6 years old, with a prevalence of 3–15% of all pediatric IBD. VEO-IBD is distinguishable from IBD in older children by a higher probability of underlying monogenic disorders or primary immunodeficiency diseases [16]. The prevalence of IBD in children has increased during the last years, such that ~ 25% of IBD-positive patients in the USA are children and adolescents [16]. With the dramatic increase in the incidence of childhood IBD compared with the other IBD categories, it is becoming more difficult for clinicians to blame monogenic mutations as the only disease-causing mechanism in VEO-IBD patients. These difficulties highlight the fact that the number of polygenic childhood IBDs with exceptional early manifestations has increased [17]. Considering the common gastrointestinal manifestations in VEO-IBD patients, distinguishing the monogenic causes (which are mostly connected to the PIDs) from the inherited polygenic causes is of great importance, especially when choosing the treatment strategy (immunosuppressants versus bone marrow transplantation) [16].

Given the overlapping gastrointestinal manifestations of the mentioned diseases and as the curable monogenic causes of VEO-IBD carry a high risk of morbidity and mortality, it is important to address the knowledge gap by reporting the rare causes of these conditions. We reported a curable cause of VEO-IBD in X-SCID patients with IBD presentation to highlight the significant role of early genetic analysis. Timely genetic assessment in patients with the same clinical presentations could be important in early diagnosis and implementing appropriate therapeutic strategies to manage severe outcomes.

## Conclusion

The initial manifestation of *IL2RG* mutation as the cause of the X-SCID could be gastrointestinal symptoms. Although this is a rare manifestation of the disease, it should not be neglected as delay in diagnosis carries a high morbidity and mortality rate. Timely genetic screening of individuals with the same clinical symptoms could help in early diagnosis and the implementation of effective treatment to handle severe outcomes.

## Data Availability

Data sharing not applicable to this article as no datasets were generated or analyzed during the current study.

## Abbreviations

Arg: Arginine
BCG: Bacille Calmette-Guérin
CADD: Combined annotation dependent depletion
CMV: Cytomegalovirus
CBC: Complete blood count
CSF: Cerebrospinal fluid
EBV: Epstein–Barr virus
FTT: Failure to thrive
GVHD: Graft-versus-host disease
G6PD: Glucose-6-phosphate dehydrogenase
GI: Gastrointestinal
HSV: Herpes simplex virus
IL2RG: Interleukin 2 receptor gamma
Ig: Immunoglobulin
IBD: Inflammatory bowel disease
Leu: Leucine
NGS: Next-generation sequencing
NK: Natural killer
PCR: Polymerase chain reaction
PID: Primary immunodeficiency disease
Trp: Tryptophan
Tregs: Regulatory T cells
VEO-IBD: Very early onset inflammatory bowel disease
WBC: White blood cells
X-SCID: X-linked severe combined immunodeficiency

## References

[1] Pai SY, Thrasher AJ (2020). Gene therapy for X-linked severe combined immunodeficiency: historical outcomes and current status. J Allergy Clin Immunol.

[2] Notarangelo LD, Giliani S, Mazza C, Mella P, Savoldi G, Rodriguez-Perez C (2000). Of genes and phenotypes: the immunological and molecular spectrum of combined immune deficiency. Defects of the gamma(c)-JAK3 signaling pathway as a model. Immunol Rev.

[3] Allenspach E, Rawlings DJ, Scharenberg AM. X-Linked Severe Combined Immunodeficiency. 2003 Aug 26 [updated 2016 Apr 14]. In: Adam MP, Ardinger HH, Pagon RA, Wallace SE, Bean LJH, Mirzaa G, Amemiya A, editors. GeneReviews^®^ [Internet]. Seattle (WA): University of Washington, Seattle; 1993–2021. 20301584

[4] Kohn DB, Hershfield MS, Puck JM, Aiuti A, Blincoe A, Gaspar HB (2018). Consensus approach for the management of severe combined immune deficiency caused by adenosine deaminase deficiency. J Allergy Clin Immunol.

[5] Noguchi M, Yi H, Rosenblatt HM, Filipovich AH, Adelstein S, Modi WS, McBride OW, Leonard WJ (1993). Interleukin-2 receptor gamma chain mutation results in X-linked severe combined immunodeficiency in humans. Cell.

[6] Jamal A, Upton J (2016). IL2RG: a series of three novel mutations with clinical manifestations. LymphoSign J..

[7] Purswani P, Meehan CA, Kuehn HS (2019). Two unique cases of X-linked SCID: a diagnostic challenge in the era of newborn screening. Front Pediatr.

[8] Estevez OA, Ortega C, Fernandez S, Aguado R, Rumbao J, Perez-Navero J, Santamaria M (2014). A novel IL2RG mutation presenting with atypical T(−)B(+)NK+ phenotype: rapid elucidation of NK cell origin. Pediatr Blood Cancer.

[9] Fuchs S, Rensing-Ehl A, Erlacher M, Vraetz T, Hartjes L (2014). Patients with T(+)/low NK(+) IL-2 receptor gamma chain deficiency have differentially-impaired cytokine signaling resulting in severe combined immunodeficiency. Eur J Immunol.

[10] Shibata F, Toma T, Wada T, Inoue M, Tone Y (2007). Skin infiltration of CD56(bright) CD16(−) natural killer cells in a case of X-SCID with Omenn syndrome-like manifestations. Eur J Haematol.

[11] Ogawa A, Watanabe T, Natsume T, Okura E, Saito S, Kato S (2021). Early-onset in ammatory bowel disease caused by mutations in the X-Linked Gene IL2RG. J Investig Allergol Clin Immunol.

[12] Gasper DJ, Tejera MM, Suresh M (2014). CD4 T-cell memory generation and maintenance. Crit Rev Immunol.

[13] Pennock ND, White JT, Cross EW, Cheney EE, Tamburini BA, Kedl RM (2013). T cell responses: naive to memory and everything in between. Adv Physiol Educ.

[14] Sharma A, Rudra D (2018). Emerging functions of regulatory T cells in tissue homeostasis. Front Immunol.

[15] Dwyer CJ, Bayer AL, Fotino C (2017). Altered homeostasis and development of regulatory T cell subsets represent an IL-2R-dependent risk for diabetes in NOD mice. Sci Signal..

[16] Ouahed J, Spencer E, Kotlarz D, Shouval DS, Kowalik M (2020). Very early onset inflammatory bowel disease: a clinical approach with a focus on the role of genetics and underlying immune deficiencies. Inflamm Bowel Dis.

[17] Kelsen JR, Russo P, Sullivan KE (2019). Early-onset inflammatory bowel disease. Immunol Allergy Clin North Am.

